# Clozapine intoxication with severe adverse effects induced by an inflammatory and infectious process: a case report

**DOI:** 10.1186/s13256-020-02660-x

**Published:** 2021-02-08

**Authors:** Emmanuel Bebawi, Leila Wakim, Maxime Doré

**Affiliations:** 1 Faculty of Medicine, University of Montreal, Roger-Gaudry Building, 2900 Edouard Montpetit Blvd, Montreal, QC H3T 1J4 Canada; 2 Department of Pharmacy, Hôpital du Sacré-Cœur de Montréal, 5400, Boulevard Gouin Ouest, Montreal, QC H4J1C5 Canada; 3 Department of Pharmacy, Jewish General Hospital, 3755 Chemin de la Côte-Sainte-Catherine, Montreal, QC H3T 1E2 Canada; 4 Faculty of Pharmacy, University of Montreal, Roger-Gaudry Building, 2900 Edouard Montpetit Blvd, Montreal, QC H3T 1J4 Canada

**Keywords:** Clozapine, Constipation, Cytochrome P450, Infection, Inflammation, Intoxication, Schizophrenia, Neutropenia

## Abstract

**Background:**

Clozapine intoxication can be life-threatening. Outside of the common drug–drug interactions, tobacco smoking, and caffeine consumption, infectious and inflammatory processes are important contributors to clozapine intoxication. Although this relationship has been reported previously, the literature is scant of proper research articles describing the presentation and management of this unpredictable interaction. Therefore, clinicians need to rely heavily on case reports describing clozapine intoxication caused by inflammation and/or infection.

**Case presentation:**

A 64-year-old Caucasian woman known for schizophrenia was brought to the emergency department (ED) with severe signs and symptoms of clozapine intoxication (general deterioration, drowsiness, neutropenia, and ileus). She was on clozapine 700 mg daily amongst other medications. The clozapine dose was stable for over 3 years, and there were no recent changes in her medications. The initial culprit was determined to be an infectious/inflammatory process of gastrointestinal origin with contribution from dehydration and constipation. Clozapine and norclozapine serum concentrations confirmed the intoxication: 1315 ng/mL and 653 ng/mL, respectively. She drastically improved with clozapine dose reduction and antibiotic therapy. She remained stable for years with clozapine 600 mg daily with stable clozapine serum levels.

**Conclusion:**

This case report illustrates the possibility of severe toxicity associated with an acute infectious and/or inflammatory process in patients on clozapine therapy. Clinicians must maintain a high level of suspicion in patients taking clozapine who develop and an infectious and/or inflammatory process. Constipation secondary to clozapine intoxication can exacerbate the initial intoxication process.

## Background

Clozapine is an atypical antipsychotic mainly used for the treatment of refractory schizophrenia. The standard daily dose of clozapine ranges from 300 to 600 mg/day, with a maximum daily dose of 900 mg [1].

Clozapine is metabolized in the liver by the cytochrome P450 (CYP) isoenzyme 1A2 into norclozapine, an active metabolite that could contribute to adverse events [2]. There is a wide interindividual variability in its metabolism, the most important factors being age, ethnicity, gender, genetic polymorphisms of CYP isoenzymes or neurotransmitter receptors, food and drink, smoking status, and drug interactions [2, 3]. It has been suggested that clozapine serum concentration monitoring may be useful in guiding therapy, optimizing therapeutic efficacy, and preventing adverse events [3].

A growing body of evidence suggests that clozapine serum concentrations may increase in patients with inflammation and/or infections, respiratory and urinary tract infections being the most frequently reported [4]. Although this relationship has been reported previously, the literature is scant of proper research articles describing the presentation and management of this unpredictable interaction. Therefore, clinicians need to rely heavily on case reports describing clozapine intoxication caused by inflammation and/or infection.

In the present report, we describe a patient who was on stable dose of clozapine for years and experienced clinically severe side effects with a decline in her general condition, associated with increased clozapine serum concentrations following sepsis.

## Case presentation

A 64-year-old Caucasian woman living in a nursing home for people with psychiatric disorders was brought to the emergency department (ED) for drowsiness, general deterioration, and hypotension, with a systolic blood pressure (sBP) of around 75–80 mmHg (normally ranging around 115–120 mmHg) at her residence. The patient’s medical history included dyslipidemia, schizophrenia which was refractory and stabilized with high dose clozapine, anorexia and bulimia with associated laxative abuse, vitamin B_12_ deficiency, overactive bladder, and an intramedullary rod in the left shin bone associated with chronic pain since 2009. According to the nursing home, she had not abused any laxatives in the last 2 years or used any recreational drugs, alcohol, or tobacco. She had no known allergies and her caffeine consumption was limited to one to five times per month. Medications were dispensed in a pillbox at the nursing home and taken under supervision: clozapine 700 mg daily, citalopram 40 mg daily, flurazepam 30 mg daily at bedtime, procyclidine 5 mg twice daily, oxybutynin 5 mg daily at bedtime, bisoprolol 2.5 mg daily, pantoprazole 40 mg daily, atorvastatin 10 mg daily, celecoxib 200 mg daily, vitamin B_12_ 1200 μg daily, calcium/vitamin D 500 mg–400 IU twice daily, and acetaminophen as needed. The clozapine dose was stable for over 3 years, and there were no recent changes in her medications. The patient was admitted to the internal medicine ward.

One to two weeks prior to the ED visit, she suffered from abdominal pain with diarrhea, vomiting, loss of appetite with decreased intake, and she gradually developed sedation and confusion. No fever was reported, but temperature was not measured at the nursing home. The review of systems was otherwise unremarkable.

**Fig. 1:**
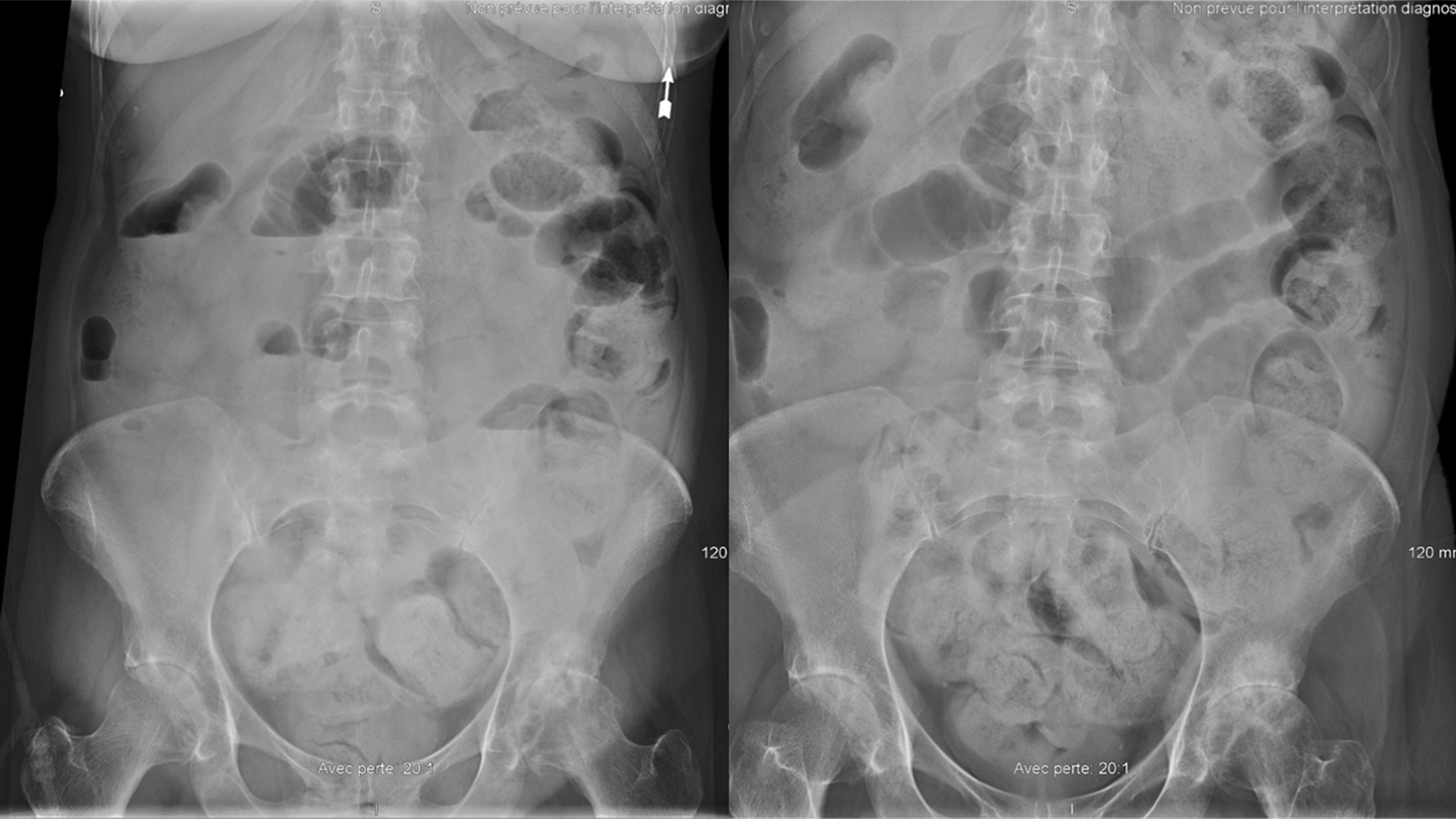
Acute abdominal series showing stools at the rectal level without any fecaloma but stercoral retention. Slight dilation of bowel loops with slight air-fluid levels

On arrival, the patient was lethargic with fluctuating confusion, sedation, loss of appetite, and abdominal pain. The diarrhea had resolved several days prior to admission. Acute constipation was suspected. The initial evaluation in the ED revealed an initial Glasgow score of 14, blood pressure of 90/59 mmHg, heart rate of 89 beats per minute (bpm), respiratory rate of 16 breaths per minute, and rectal temperature of 37.5 °C. Shortly after, a fever was recorded (38.4 °C orally). Physical examination was unremarkable and did not offer any substantial information. Laboratory tests revealed hypokalemia (3.2 mmol/L), elevated C-reactive protein (CRP) (320 mg/L), acute kidney injury (creatinine 115 µmol/L, baseline 85 µmol/L), anemia (hemoglobin 112 g/L), lymphopenia (0.5 × 10^9^ cells/L), and neutropenia (1.5 × 10^9^ cells/L) with a normal leukocyte count (4.6 × 10^9^ cells/L). Peripheral blood smear showed Döhle bodies and toxic granulations. Six days earlier, the neutrophil count was 3.7 × 10^9^ cells/L and hemoglobin concentration was 122 g/L. A chest radiograph was completed and appeared normal. A cerebral computed tomography (CT) scan without contrast was normal without any signs of acute intracranial process. The acute abdominal series (Fig. 1) showed light dilated loops with small air-fluid levels and diffuse stercoral retention with numerous feces formed at the rectal level without actual fecal impaction. She was successfully treated with an enema, and oxybutynin was discontinued. Developing intestinal paralytic ileus was suspected. Her blood pressure and renal function returned to normal following rehydration by intravenous (IV) fluids. Hypokalemia was corrected with potassium supplements. From this point forward, the patient’s blood pressure and heart rate remained stable during the hospital stay, at around 110–120/55–65 mmHg and 100–115 bpm, respectively. The first diagnostic hypothesis was sepsis from a gastrointestinal origin.

The next day (day 2) another febrile episode of 39.1 °C was recorded. The precise source of the infection was still unknown, but infectious colitis with possible bacterial translocation was suspected. The patient was still drowsy, had nausea, and her abdominal pain was still present. Toxicology screen was unremarkable. Concomitant drug intoxication by clozapine, citalopram, or flurazepam was considered. Blood samples were drawn to measure serum levels of clozapine/norclozapine, and CRP. The CRP level was 148 mg/L. Subsequently, piperacillin-tazobactam 3.375 g IV every 6 hours was empirically started. Clozapine, citalopram, and procyclidine doses were empirically decreased (400 mg daily, 20 mg daily, and 2.5 mg twice daily, respectively) and flurazepam was discontinued. An abdominal and pelvic CT scan revealed a thickened sigmoid colon without distension or signs of inflammation and presence of some of the small bowel loops being distended and interpreted by the radiologist as early signs of occlusion. A colonoscopy was performed 3 days later and was normal. Completed blood and stool cultures were negative.

On day 5, the results for clozapine and norclozapine serum concentrations (drawn on day 2) were available: 1315 ng/mL and 653 ng/mL, respectively. The high clozapine serum concentration, taken in the context of general deterioration, altered mental status, neutropenia, and ileus, confirmed the hypothesis of clozapine intoxication. The general condition of the patient had improved, including gastrointestinal transit, in the days following the reduction of clozapine, citalopram, and procyclidine doses, discontinuation of flurazepam, and antibiotic initiation. A total antibiotic course of 7 days was completed. Clozapine dose was raised to 500 mg daily as the patient’s condition improved. In order to maintain clozapine therapy despite the neutropenia and to avoid issues with the clozapine regulatory program, the patient received one subcutaneous dose of filgrastim 300 μg while the neutrophil and leukocyte counts were 1.6 × 10^9^ cells/L and 2.5 × 10^9^ cells/L, respectively.

On day 6, the CRP decreased (18 mg/L) and the neutrophil count increased to 21.5 × 10^9^ cells/L. On day 7, clozapine dose was increased to 550 mg daily, citalopram dose was increased to 30 mg daily, and procyclidine was discontinued. At this point, the clozapine and norclozapine serum concentrations had decreased to 894 ng/mL and 443 ng/mL, respectively. At hospital discharge, on day 9, the clozapine and norclozapine serum concentrations were 859 ng/mL and 582 ng/mL respectively, while the neutrophil count was back to normal (5.2 × 10^9^ cells/L). The patient was discharged with a clozapine dose of 600 mg daily.

Clozapine and norclozapine serum concentrations obtained 4 days after hospital discharge were 831 ng/mL and 582 ng/mL, respectively, and the neutrophil count was 3.6 × 10^9^ cells/L. Two years later, the patient remained clinically stable on clozapine 600 mg daily with a clozapine and norclozapine serum concentrations of 828 ng/mL and 685 ng/mL, respectively.

## Discussion and conclusions

This case highlights the importance of understanding clozapine metabolism. Clozapine is mainly metabolized by CYP1A2 (70%), and to a lesser extent by CYP2D6, 3A4, 2C9, and 2C19 [3, 4]. Modifying CYP1A2 activity has an important impact, as seen with tobacco smoking which induces metabolism resulting in reduced clozapine serum concentrations, and caffeine consumption which inhibits metabolism, resulting in increased clozapine serum concentrations [3]. Rises in concentrations can be the result of drug–drug interactions with strong CYP1A2 inhibitors, such as ciprofloxacin [3]. In the present case, the patient was a non-smoker, rarely consumed caffeine, and no drug interaction was identified. A clozapine overdose was unlikely as medication was dispensed in a pillbox and taken under supervision.

**Fig. 2:**
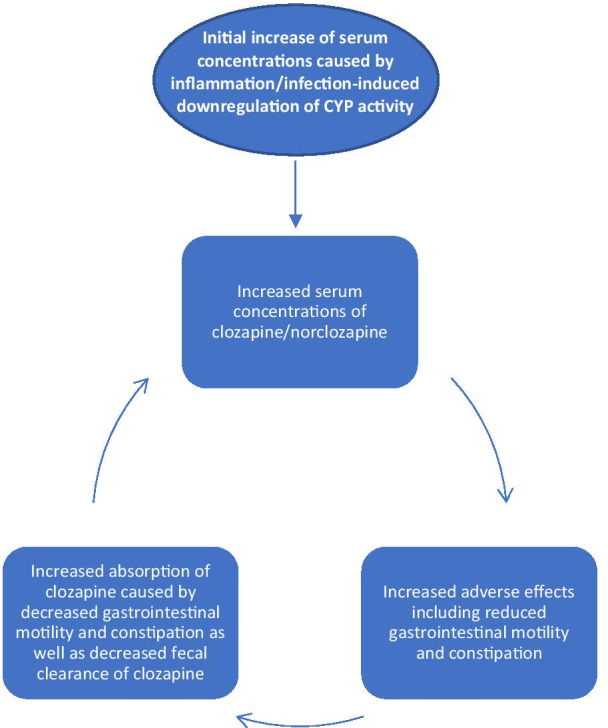
Schematic proposed explanation for clozapine intoxication

In our case, an acute infectious/inflammatory process was believed to have sparked clozapine intoxication, with dehydration and added constipation exacerbating clozapine toxicity by increasing its absorption (Fig. 2). Prior to hospitalization, the patient’s condition was stable with her pharmacotherapy and was well tolerated for years. She had regular complete blood count monitoring as part of the clozapine regulatory program, showing no signs of toxicity. She developed acute gastroenteritis/colitis, which was determined as the initiating factor to acute clozapine intoxication. She progressively developed adverse effects associated with clozapine: sedation, confusion, neutropenia, and ileus. Confusion and sedation lead to reduced fluid intake contributing to dehydration and general deterioration. Furthermore, extensive workup was conducted to rule out other causes of mental status change but was unremarkable. The psychiatrist’s evaluation did not show deterioration of her schizophrenia, nor did it highlight delirium.

Serum concentration of clozapine, taken shortly after her admission, confirmed intoxication with levels exceeding 1000 ng/mL. A serum concentration range of 350–400 ng/mL is a reasonable target for patients with schizophrenia, but great variation in symptomatic response and side effects is observed [3, 5]. Furthermore, some authors showed that in the absence of clinical improvement at these serum concentrations, clozapine should be further increased until intolerable side effects occur or a maximum dose of 900 mg/daily is reached [3]. A definite clozapine serum concentration associated with toxicity remains unclear. However, it is suggested that adverse effects are more likely to occur with serum levels above 600–1000 ng/mL [3].

The finding of diffuse stercoral retention was explained by the concomitant use of anticholinergic drugs in addition to clozapine intoxication. The temporal relationship, in addition to a normal colonoscopy, led us to favor the latter into explaining acute constipation with radiographic signs of ileus. Constipation and decreased gastrointestinal motility has been correlated with increased clozapine serum levels as it may reflect its antimuscarinic activity [6, 7]. Constipation likely contributed to exacerbation of intoxication by increasing drug absorption and decreasing fecal drug elimination.

In our case, there was very little clinical, radiological, or laboratory evidence of an infectious/inflammatory process, other than fever and elevated CRP levels. This can be explained by clozapine toxicity as it may prevent increase of white blood counts (notably neutrophil count) and development of fever [7]. Therefore, clinical hallmarks of infection may be subtle or even absent, with elevated CRP being the only clue [7]. As such, CRP levels should be measured in the context of clozapine intoxication. Furthermore, an elevated CRP in patients taking clozapine should prompt a thorough evaluation in finding the cause and aggressively seeking signs and symptoms of clozapine intoxication. The patient’s condition improved, and CRP level decreased with antibiotic therapy.

Resolution of overall toxicity occurred in a temporal relationship after decreasing clozapine dose, which correlated with decreased serum concentrations of clozapine and norclozapine. The application of the Naranjo Adverse Drug Reaction Probability Scale resulted in a score of 7, indicating that clozapine intoxication was probable (one point for previous conclusive reports of clozapine toxicity, two points because the adverse event appeared after the suspected drug was administered, two points because there were no known alternative causes, one point because the drug was detected in the serum in concentrations known to be toxic, and one point because the reaction became less severe after the dose was decreased).

The mechanism behind increased clozapine serum concentrations induced by an infectious/inflammatory process is not clearly understood. Inflammation affects metabolic pathways by altering the expression of CYP via inflammation-induced downregulation of CYP activity [8]. Infection or inflammation can decrease metabolic clearance of CYP substrates by 20–70% [9], notably reducing CYP1A2 expression by up to 90% [4]. The proposed mechanism is a direct loss of CYP450 gene transcription via cytokine-induced transcriptional depression resulting in downregulation of CYP activities and enzyme synthesis [10, 11]. The cytokines implicated in decreased expression of CYP1A2 are interleukin-6, tumor necrosis factor-α, and interleukin-1β [9–11]. Modification at the post-translational level is also implicated, with an increase in CYP degradation or a change in their enzymatic activity following cytokine release [8, 10].

A study investigated the correlation between pathological CRP levels and serum concentrations of clozapine, quetiapine, and risperidone [12]. Elevated CRP levels were associated with significantly elevated serum concentrations for clozapine (*P* < 0.01) and risperidone (*P* < 0.01), quetiapine having only a trend in increased serum concentrations (*P* = 0.05) [12]. The authors concluded that in patients with signs of infection/inflammation with increased CRP levels, therapeutic drug monitoring is recommended in order to minimize the risk of intoxication [12].

Clozapine toxicity secondary to inflammation/infection is variable from one patient to another. This can be attributed to alpha-1-glycoprotein (AGP), an acute phase protein to which 95% of clozapine is bound [4]. During infection/inflammation, AGP increases several-fold, thereby increasing the concentration of clozapine along with AGP leading to elevation of total drug level [4, 13]. However, the free drug level is not necessarily increased, explaining the absence of clinical toxicity in some patients [4, 13]. AGP increase might not be sufficient in all cases to prevent clozapine intoxication.

In the setting of an acute infectious/inflammatory process, the optimal clozapine dose reduction is unknown. It has been suggested that at least a 50% dose reduction may be required to avoid toxicity [14]. Clozapine serum concentration monitoring is used to confirm clinical intoxication by comparing it to a prior tolerated baseline serum level. However, in the scenario of evident signs and symptoms of clozapine toxicity, doses should be reduced empirically to ensure patient safety, even if clozapine serum levels are not available. As stated earlier, not all elevated serum levels will occur with clinical signs of toxicity. In these cases, maintaining the patient’s current dose with close monitoring for adverse effects and repeating clozapine serum levels after the acute infectious/inflammatory process resolves may be appropriate.

This case report highlights the possibility of severe toxicity associated with an acute infectious and/or inflammatory process in patients on clozapine therapy. This process is insidious, and it is therefore imperative that these patients be closely monitored for clozapine toxicity. It is essential that ileus and constipation be identified and treated quickly as they can be secondary to the initial intoxication process and contribute in perpetuating a vicious circle of intoxication by increasing drug absorption and decreasing fecal elimination of clozapine. CRP and clozapine serum levels are useful guiding tools for identifying and monitoring this phenomenon. Further research is warranted.

## Data Availability

Not applicable.
